# Leptin modulates electrophysiological characteristics and isoproterenol-induced arrhythmogenesis in atrial myocytes

**DOI:** 10.1186/1423-0127-20-94

**Published:** 2013-12-20

**Authors:** Yung-Kuo Lin, Yao-Chang Chen, Jen-Hung Huang, Yenn-Jiang Lin, Shiang-Suo Huang, Shih-Ann Chen, Yi-Jen Chen

**Affiliations:** 1 Division of Cardiovascular Medicine, Department of Internal Medicine, Wan Fang Hospital, Taipei Medical University, 111, Hsin-Lung Road, Sec. 3, Taipei, Taiwan; 2 Graduate Institute of Clinical Medicine, College of Medicine, Taipei Medical University, Taipei, Taiwan; 3 Department of Biomedical Engineering, National Defense Medical Center, Taipei, Taiwan; 4 National Yang-Ming University, School of Medicine, Division of Cardiology and Cardiovascular Research Center, Veterans General Hospital-Taipei, Taipei, Taiwan; 5 Department of Pharmacology and Institute of Medicine, Chung Shan Medical University, Taichung, Taiwan; 6 Department of Pharmacy, Chung Shan Medical University Hospital, Taichung, Taiwan

**Keywords:** Adipokines, Atrial fibrillation, Epicardial fat, Leptin, Obesity

## Abstract

**Background:**

Obesity is an important risk factor for atrial fibrillation (AF). Leptin is an important adipokine. However, it is not clear whether leptin directly modulates the electrophysiological characteristics of atrial myocytes.

**Results:**

Whole cell patch clamp and indo-1 fluorescence were used to record the action potentials (APs) and ionic currents in isolated rabbit left atrial (LA) myocytes incubated with and without (control) leptin (100 nM) for 1 h to investigate the role of leptin on atrial electrophysiology. Leptin-treated LA myocytes (n = 19) had longer 20% of AP duration (28 ± 3 vs. 21 ± 2 ms, p < 0.05), but similar 50% of AP duration (51 ± 4 vs. 50 ± 3 ms, p > 0.05), and 90% of AP duration (89 ± 5 vs. 94 ± 4 ms, p > 0.05), as compared to the control (n = 22). In the presence of isoproterenol (10 nM), leptin-treated LA myocytes (n = 21) showed a lower incidence (19% vs. 54.2%, p < 0.05) of delayed afterdepolarization (DAD) than the control (n = 24). Leptin-treated LA myocytes showed a larger sodium current, but a smaller ultra-rapid delayed rectifier potassium current, and sodium-calcium exchanger current than the control. Leptin-treated and control LA myocytes exhibited a similar late sodium current, inward rectifier potassium current, transient outward current and L-type calcium current. In addition, the leptin-treated LA myocytes (n = 38) exhibited a smaller intracellular Ca^2+^ transient (0.21 ± 0.01 vs. 0.26 ± 0.01 R410/485, p < 0.05) and sarcoplasmic reticulum Ca^2+^ content (0.35 ± 0.02 vs. 0.43 ± 0.03 R410/485, p < 0.05) than the control LA myocytes (n = 42).

**Conclusions:**

Leptin regulates the LA electrophysiological characteristics and attenuates isoproterenol-induced arrhythmogenesis.

## Background

Obesity is an independent risk factor for the genesis of AF [1-4]. Atrial fibrillation (AF) is the most prevalent cardiac arrhythmia in clinical practice which can induce cardiac dysfunction and stroke [5]. Obesity increase the prevalence of hypertension, ischemic heart disease, congestive heart failure, and ventricular dysfunction, which can contribute to the development of AF [6-9]. In addition, epicardial fat can change cardiac electrophysiology by increasing inflammation, mechano-electrical regulation and adipocyte-myocytes interaction [10]. Adipose tissues can produce many adipokines, which may have electrophysiolgical effects through changing action potential morphology, ionic profiles and contractility in the atrium [11,12]. However, it is not clear which adipokine is responsible for the cardiac effects of adipose tissues.

Leptin is a peptide hormone that is expressed in adipose tissue, and that regulates body weight through the inhibition of food intake and promotion of energy expenditure [13,14]. However, leptin can also be produced in the heart, and function in an autocrine and paracrine manner [15,16]. Leptin plasma concentrations are increased in insulin-resistant states, such as obesity and hypertension [17-19]. Similarly, leptin concentrations are elevated in patients with heart disease such as ischemic heart disease and congestive heart failure [20,21]. However, clinical studies have yielded inconsistent results on the association of leptin with cardiovascular disease [20,22]. Evidences have indicated that leptin may have either adverse or beneficial effects on the heart and the vascular system [23-27]. In addition, leptin is considered a novel link between obesity, diabetes, cardiovascular risks and ventricular hypertrophy [28]. Leptin signaling has been shown to contribute to atrial fibrosis and angiotensin II-evoked AF [29]. These findings suggest that leptin may play a critical role in cardiac electrophysiology. The purpose of this study was to investigate the effect of leptin on the electrophysiological characteristics of atria myocytes and its role in atrial arrhythmogenesis.

## Methods

### Isolation of single LA myocytes

All animal experimental procedures were approved by the Institutional Animal Care and Use Committee (IACUC) at Taipei Medical University (Protocol Number LAC-100-0127) and conform to the institutional Guide for the Care and Use of Laboratory Animals and the Guide for the Care and Use of Laboratory Animals published by the US National Institutes of Health (NIH Publication No. 85-23, revised 1996). Male rabbits (1 to 2 kg) were intravenously injected with sodium pentobarbital (100 mg/kg). After anesthetization, the hearts were immediately removed and mounted on a Langendorff apparatus to perform retrograde perfusion of oxygenated normal Tyrode’s solution at 37°C containing (in mM): NaCl 137, KCl 5.4, CaCl_2_ 1.8, MgCl_2_ 0.5, HEPES 10 and glucose 11; adjusted to pH 7.4 with NaOH. After the hearts were cleaned of blood, the perfusate was replaced with oxygenated Ca^2+^-free Tyrode’s solution containing 300 units/ml collagenase type I (Sigma Chemical, St, Louis, MO) and 0.25 units/ml protease type XIV (Sigma) for approximately 8 to12 min. The left atrium (LA) was excised and gently shaken in 50 ml of Ca^2+^-free oxygenated Tyrode’s solution until single cardiomyocytes were obtained. The solution was then gradually changed to normal oxygenated Tyrode’s solution. The myocytes were allowed to stabilize in the bath for at least 30 min before the experiments.

### Electrophysiological study

A whole-cell patch clamp was performed in the LA myocyte with and without (control) incubation of leptin (100 nM) for 1 h using an Axopatch 1D amplifier (Axon Instruments, CA, USA) at 35±1°C as described previously [11,30,31]. Borosilicate glass electrodes (o.d., 1.8 mm) were used, with tip resistances of ~ approximately 3 to 5 MΩ. Before formation of the membrane-pipette seal, the tip potentials were zeroed in Tyrode’s solution. Junction potentials between the bath and pipette solution (9 mV) were corrected for action potential (AP) recordings. APs were recorded in the current-clamp mode, and ionic currents were measured in the voltage-clamp mode. A small hyperpolarizing step from a holding potential of -50 mV to a testing potential of -55 mV for 80 ms was delivered at the beginning of each experiment. The area under the capacitive currents was divided by the applied voltage step to obtain the total cell capacitance. Normally, 60%-80% series resistance (R_s_) was electronically compensated. The resting membrane potential (RMP) was measured during the period between the last repolarization and the onset of the subsequent AP. The AP amplitude (APA) was obtained from the measurement of RMP to the peak of the AP depolarization. AP durations were measured at 20% (APD_20_), 50% (APD_50_), and 90% (APD_90_) repolarization of the amplitude at a driven rate of 1 Hz. DADs (delayed afterdepolarizations) were defined as the presence of a spontaneous depolarization of the impulse after full repolarization had occurred.

Micropipettes were filled with a solution containing the following (in mM): CsCl 130, MgCl_2_ 1, Mg_2_ATP 5, HEPES 10, EGTA 10, NaGTP 0.1, and Na_2_ phosphocreatine 5, titrated to a pH of 7.2 with CsOH for the experiments on the L-type calcium current (*I*_Ca-L_); containing (in mM) CsCl 133, NaCl 5, EGTA 10, Mg_2_ATP 5, TEACl 20, and HEPES 5 (pH 7.3 with CsOH) for the sodium current (*I*_Na_); containing (in mM) 10 NaCl, 130 CsCl, 5 EGTA, 5 HEPES, 5 glucose, and 5 ATP-Mg for the late sodium current (*I*_Na-Late_); containing (in mM) NaCl 20, CsCl 110, MgCl_2_ 0.4, CaCl_2_ 1.75, tetraethylammonium chloride (TEACl) 20, BAPTA 5, glucose 5, Mg_2_ATP 5, and HEPES 10, titrated to a pH of 7.25 with CsOH for the experiments on sodium-calcium exchanger (NCX) current; and containing (in mM) KCl 20, K aspartate 110, MgCl_2_ 1, Mg_2_ATP 5, HEPES 10, EGTA 0.5, LiGTP 0.1, and Na_2_ phosphocreatine 5, titrated to a pH of 7.2 with KOH for the experiments on the AP and potassium currents.

The *I*_Na_ was recorded during depolarization from a holding potential of -120 mV to testing potentials ranging from -90 to 0 mV in 10-mV steps for 40 ms at a frequency of 3 Hz at room temperature (25 ± 1°C) with an external solution containing (in mM): NaCl 5, CsCl 133, MgCl_2_ 2, CaCl_2_ 1.8, nifedipine 0.002, HEPES 5 and glucose 5 with a pH of 7.3. The *I*_Na-Late_ was recorded at room temperature with an external solution containing (in mM): 140 NaCl, 5 CsCl, 2.0 MgCl_2_, 1.8 CaCl_2_, 5 HEPES, 5 glucose, and 0.002 of nicardipine. The amplitude of the *I*_Na-Late_ was measured at a voltage of -20 mV as the mean current amplitude between 200 and 250 ms after the membrane was depolarized by a 2,000-ms pulse from -140 to -20 mV.

The *I*_Ca-L_ was measured as an inward current during depolarization from a holding potential of −50 mV to testing potentials ranging from -40 to +60 mV in 10-mV steps for 300 ms at a frequency of 0.1 Hz using a perforated patch clamp with amphotericin B. The NaCl and KCl in the external solution were replaced with tetraethylammonium chloride and CsCl, respectively.

The transient outward current (*I*_to_) was studied with a double-pulse protocol. A 30-ms prepulse from −80 to −40 mV was used to inactivate the sodium channels, followed by a 300-ms test pulse to +60 mV in 10-mV steps at a frequency of 0.1 Hz. CdCl_2_ (200 μM) was added to the bath solution to inhibit *I*_Ca-L_. The *I*_to_ was measured as the difference between the peak outward current and steady-state current. The ultra-rapid delayed rectifier potassium current (*I*_Kur_) was studied with a double-pulse protocol, consisting of a 100-ms depolarizing pre-pulse to +40 mV from a holding potential of -50 mV, followed by 150-ms voltage steps from -40 to +60 mV in 10 mV increments at room temperature to provide an adequate temporal resolution. The *I*_Kur_ was measured as 4-aminopyridine (1 mM) sensitive currents.

The inward rectifier potassium current (*I*_K1_) was activated from -40 mV to test potentials ranging from -20 to -120 mV in 10-mV steps for 1 s at a frequency of 0.1 Hz under the infusion of CdCl_2_ (200 μM) and 4-aminopyridine (2 mM) in the bath solution. The amplitudes of the *I*_K1_ were measured as 1 mM barium-sensitive currents.

The NCX current was elicited by test potentials between -100 to +100 mV from a holding potential of −40 mV for 300 ms at a frequency of 0.1 Hz. The amplitudes of the NCX current were measured as 10 mM nickel-sensitive currents. The external solution (in mM) consisted of NaCl 140, CaCl_2_ 2, MgCl_2_ 1, HEPES 5 and glucose 10 with a pH of 7.4 and contained strophanthidin (10 μM), nitrendipine (10 μM) and niflumic acid (100 μM).

### Measurement of the changes in the intracellular calcium concentration

The intracellular Ca^2+^ (Ca^2+^_*i*_) was recorded using a fluorimetric ratio technique (indo-1 fluorescence) in an isolated single leptin-treated and control LA myocyte, as described previously [31,32]. The fluorescent indicator indo-1 was loaded by incubating the myocytes at room temperature for 20–30 min with 10 μM of indo-1/AM (Sigma Chemical, St Louis, MO, USA). LA myocytes were then perfused with Tyrode's solution at 35 ± 1°C for at least 20 min to wash out the extracellular indicator and to allow for the intracellular de-esterification of the indo-1. The background and cell autofluorescence were canceled out by zeroing the output of the photomultiplier tubes using cells without indo-1 loading. A UV light of 360 nm with a monochromator was used for the excitation of the indo-1 from a xenon arc lamp controlled by the microfluorimetry system (OSP100-CA, Olympus, Tokyo, Japan) and the excitation light beam was directed into an inverted microscope (IX-70, Olympus, Tokyo, Japan). The emitted fluorescence signals from the indo-1/AM loaded myocytes were digitized at 200 Hz. The ratio of the fluorescence emission at 410 and 485 nm (R410/485) was used as the index of the Ca^2+^_*i*_. This approach avoided any uncertainties resulting from the calibration of the fluorescent Ca^2+^ indicators. The Ca^2+^_*i*_ transients were measured during a 2 Hz field stimulation with 10 ms square-wave pulses at double threshold strength and were calculated from the difference of the peak systolic and diastolic Ca^2+^_*i*_ transients. The fluorescence ratio data were processed and stored on a computer using the relevant software (OSP-SFCA, Olympus, Tokyo, Japan). The sarcoplasmic reticulum (SR) Ca^2+^ content was estimated by adding 20 mM of caffeine after electric stimulation at 2 Hz for at least 30 s. The total SR Ca^2+^ content was measured from the peak amplitude of the caffeine-induced Ca^2+^_*i*_ transients. We also measured the SR Ca^2+^ content by integrating the NCX current from rapidly adding 20 mM of caffeine to the cells during rest with the membrane potential clamped to -40 mV. The time integral of the NCX current was converted to amoles of Ca^2+^ released from the SR [31].

### Statistical analysis

All continuous variables are expressed as the mean ± SEM. The differences between the control and the leptin-treated LA myocytes were compared using the Mann-Whiteney rank sum test or unpaired *t*-test depending on the outcome of the normality test. A p value of less than 0.05 was considered statistically significant.

## Results

### Effects of leptin on the electrical activity of LA myocytes

**Figure 1:**
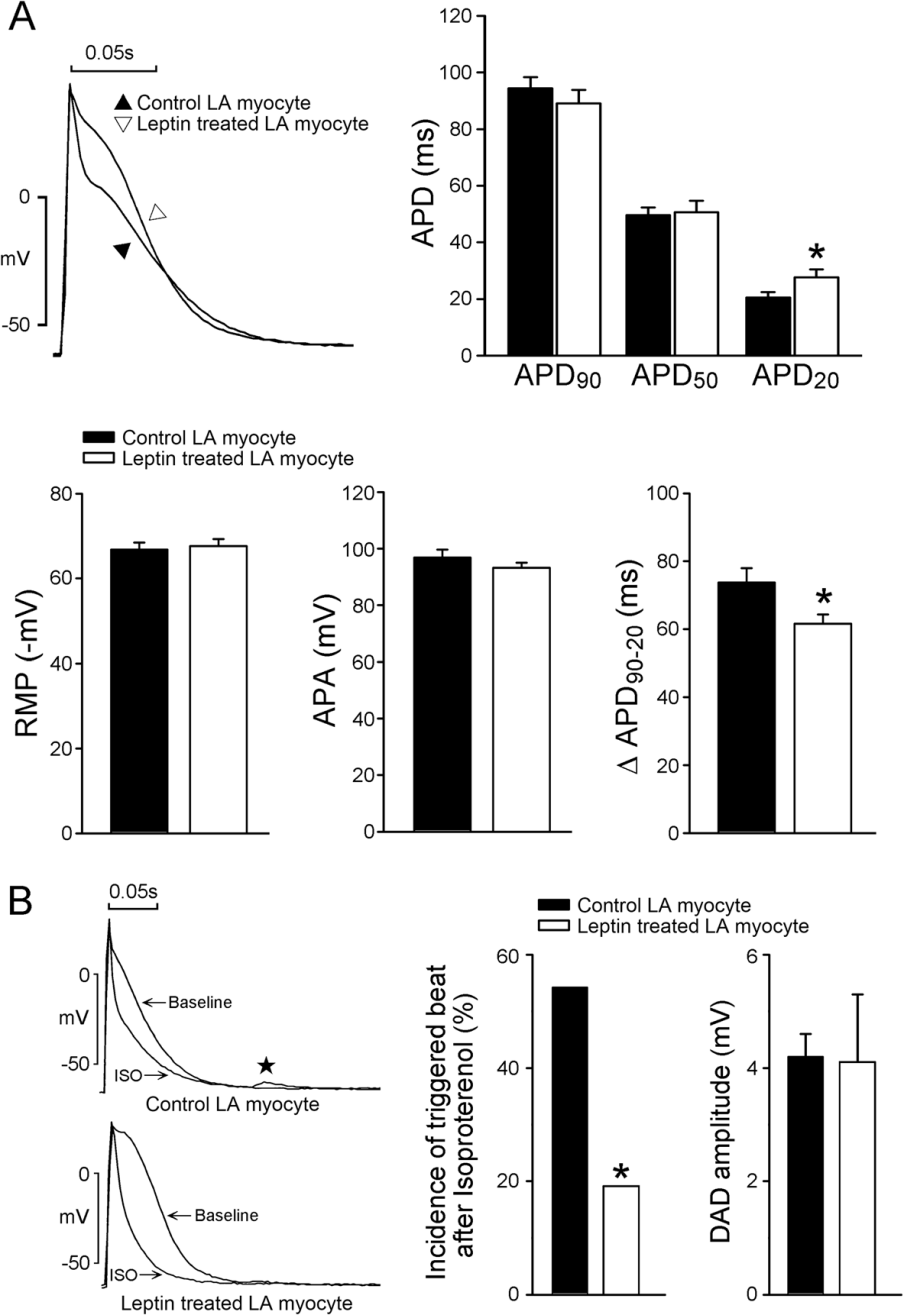
**Effect of leptin on the left atrial (LA) electrical activity and effects of isoproterenol on control and leptin-treated left atrial (LA) myocytes. A**. Examples and average data of the action potential from control (n = 22) LA myocytes and leptin-treated LA myocytes (n = 19). Leptin-treated LA myocytes had longer APD_20_. **B**. Examples of isoproterenol-induced DAD (★) from control and leptin-treated LA myocytes. The incidence of isoproterenol-induced DADs from control (n = 24) and leptin-treated (n = 21) LA myocytes. RMP resting membrane potential, APA action potential amplitude, APD_90_ 90% action potential duration, APD_50_ 50% action potential duration, APD_20_ 20% action potential duration. *p < 0.05 versus control LA myocytes.

Figure 1A shows the AP morphology from the control and the leptin-treated LA myocytes. The leptin-treated LA myocytes had longer APD_20_, but similar APD_50_, APD_90_, and RMP, compared to the control. Moreover, the difference between APD_90_ and APD_20_ (∆APD_90-20_) was smaller in the leptin-treated LA myocytes than in the control LA myocytes. In the presence of isoproterenol (10 nM), leptin- treated LA myocytes exhibited a lower incidence of DADs than the control myocytes (Figure 1B).

### Effects of leptin on the membrane currents of LA myocytes

**Figure 2:**
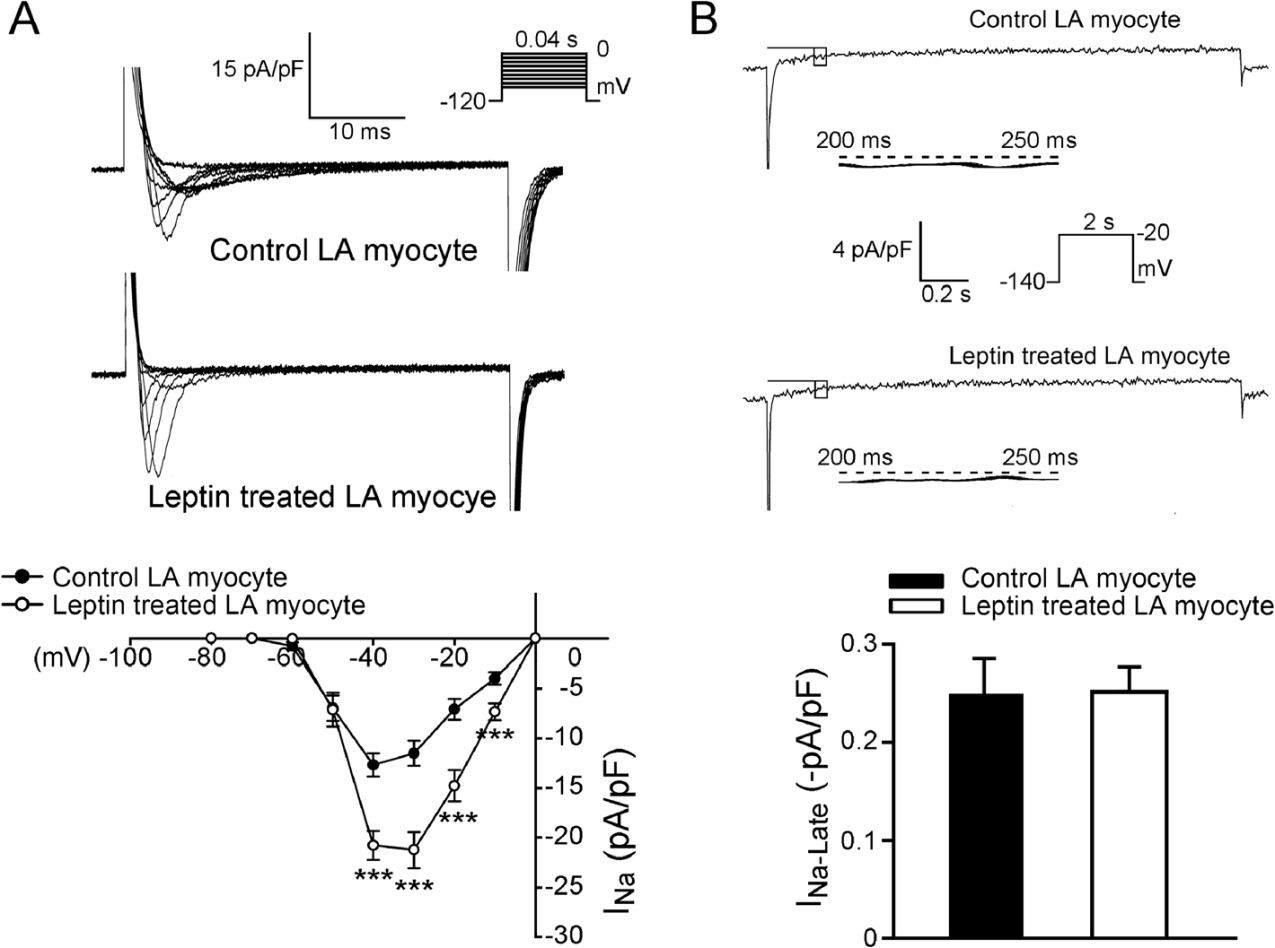
**Effects of leptin on sodium current (*I***_**Na**_**) and late sodium current (*I***_**Na-Late**_**) in left atrial (LA) myocytes. A**. Examples and the I–V relationship of the *I*_Na_ from control (n = 21) and leptin-treated (n = 14) LA myocytes. The insets in the current traces show the various clamp protocols. **B**. Examples and the average data of the *I*_Na-Late_ in control (n = 8) and leptin-treated myocytes (n = 8). ***p < 0.005 versus control LA myocytes.

**Figure 3:**
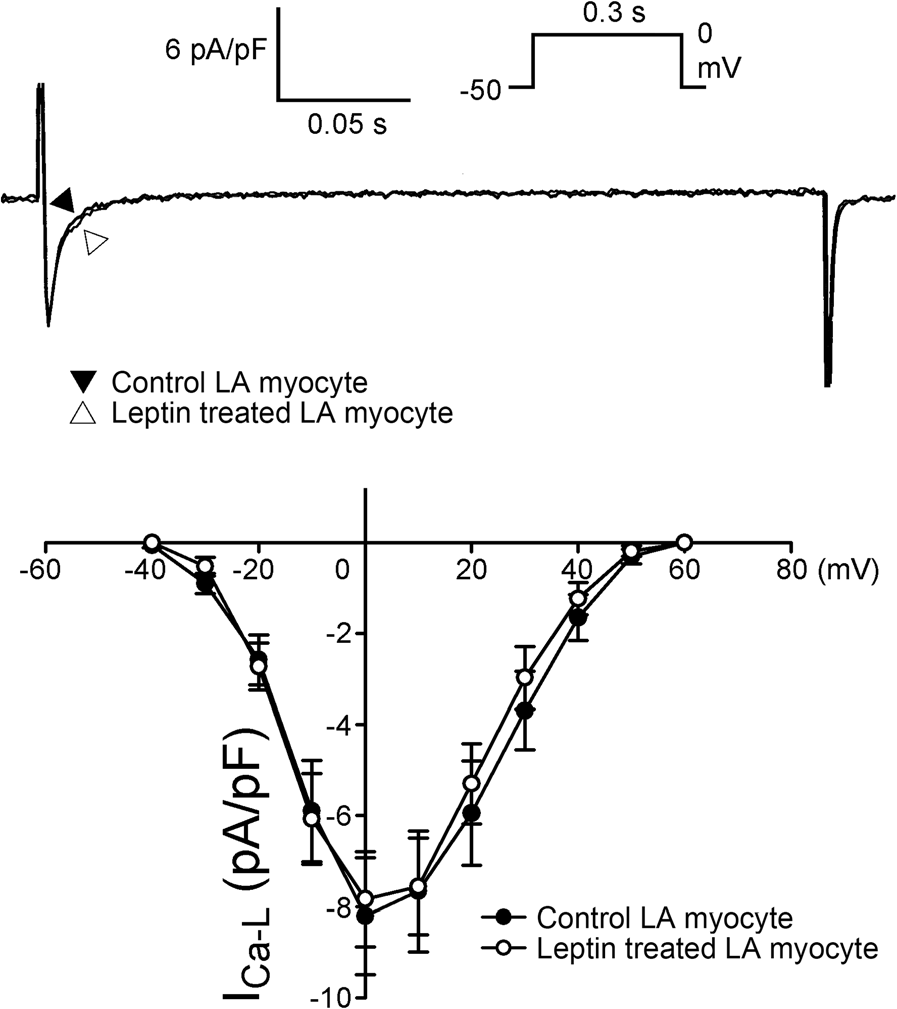
**Effects of leptin on the L-type calcium channel (*I***_**Ca-L**_**) in left atrial (LA) myocytes.** The current traces and I–V relationship of *I*_Ca-L_ from control (n = 14) and leptin-treated (n = 10) LA myocytes. The insets in the current traces show the various clamp protocols.

**Figure 4:**
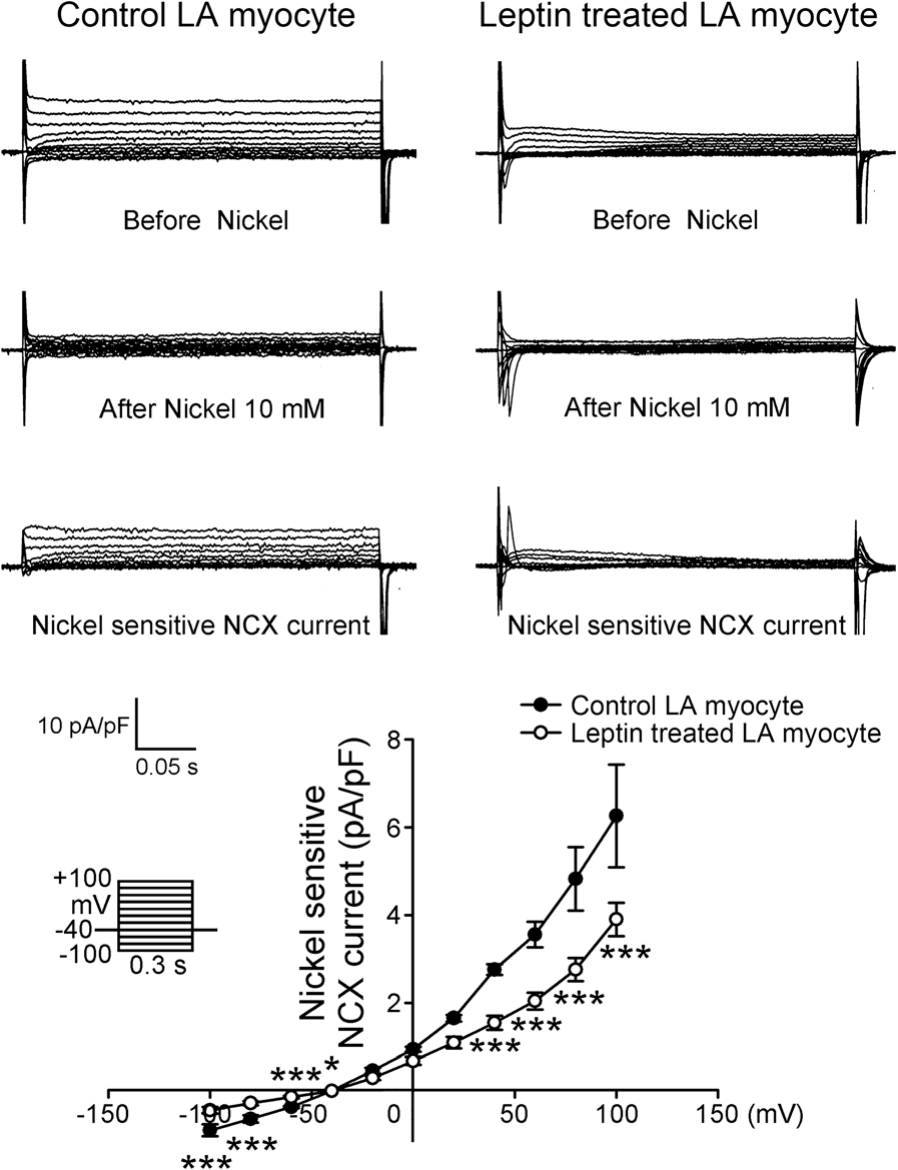
**The current tracings, I–V relationship of the nickel-sensitive Na**^**+**^**–Ca**^**2+ **^**exchanger (NCX) currents in the control (n = 14) and leptin-treated LA myocytes (n = 14).** The leptin-treated LA myocytes have significantly smaller nickel-sensitive NCX currents than the control LA myocytes do. The insets in the current traces show the various clamp protocols. *p < 0.05; ***p < 0.005 versus control LA myocytes.

Compared to the control LA myocytes, the leptin-treated LA myocytes exhibited larger *I*_Na_ (Figure 2A) with a 46% increase of peak current (elicited from -120 to -40 mV). Both the control and the leptin-treated LA myocytes exhibited similar *I*_Na-Late_ (Figure 2B) and similar *I*_Ca-L_ (Figure 3). The forward and reverse mode of NCX currents were smaller in the leptin-treated LA myocytes (Figure 4) than in the control myocytes with a 37.6% decrease in peak current (elicited from -40 to -100 mV) in the forward mode and a 51% decrease in peak current (elicited from -40 to 100 mV) in the reverse mode.

**Figure 5:**
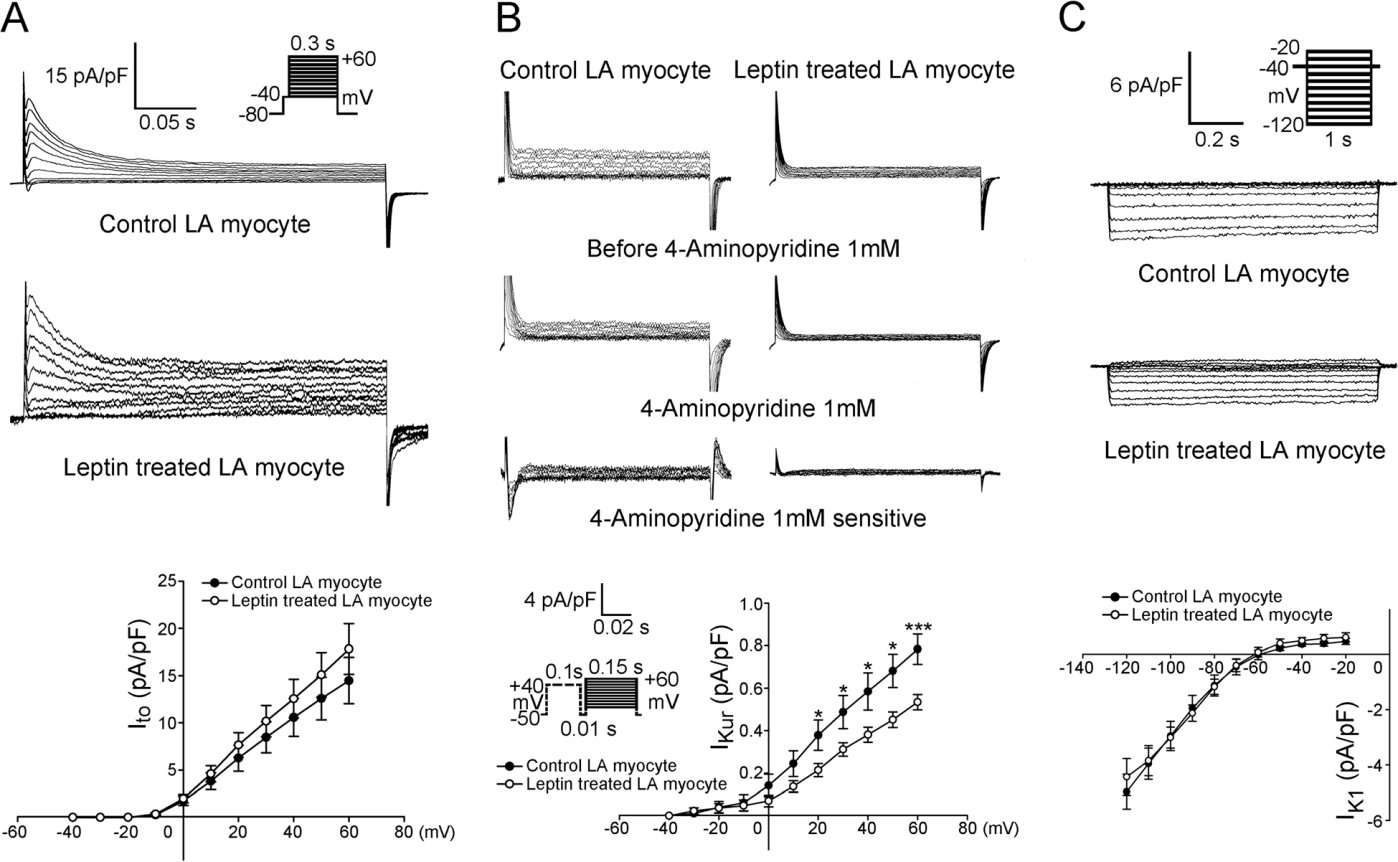
**Effects of leptin on the transient outward current (*I***_**to**_**), the ultra-rapid delayed rectifier potassium current (*I***_**Kur**_**) and the inward rectifier potassium current (*I***_**K1**_**) in left atrial (LA) myocytes. A**. Examples and I–V relationship of the *I*_to_ from control (n = 11) and leptin-treated (n = 16) LA myocytes. **B**. Examples and I–V relationship of the *I*_Kur_ from control (n = 12) and leptin-treated (n = 14) LA myocytes. **C**. Examples and the I–V relationship of the *I*_K1_ from control (n = 8) and leptin-treated (n = 10) LA myocytes. The inset in the current traces shows the clamp protocol. *p < 0.05; ***p < 0.005 versus control LA myocytes.

As compared to the control myocytes, the leptin-treated LA myocytes exhibited similar *I*_to_, but smaller *I*_Kur_ with a 32% decrease of peak current (elicited from -50 to 60 mV) (Figure 5A and 5B). The leptin-treated and control myocytes exhibited similar *I*_K1_ (Figure 5C).

### Effects of leptin on calcium handling of LA myocytes

**Figure 6:**
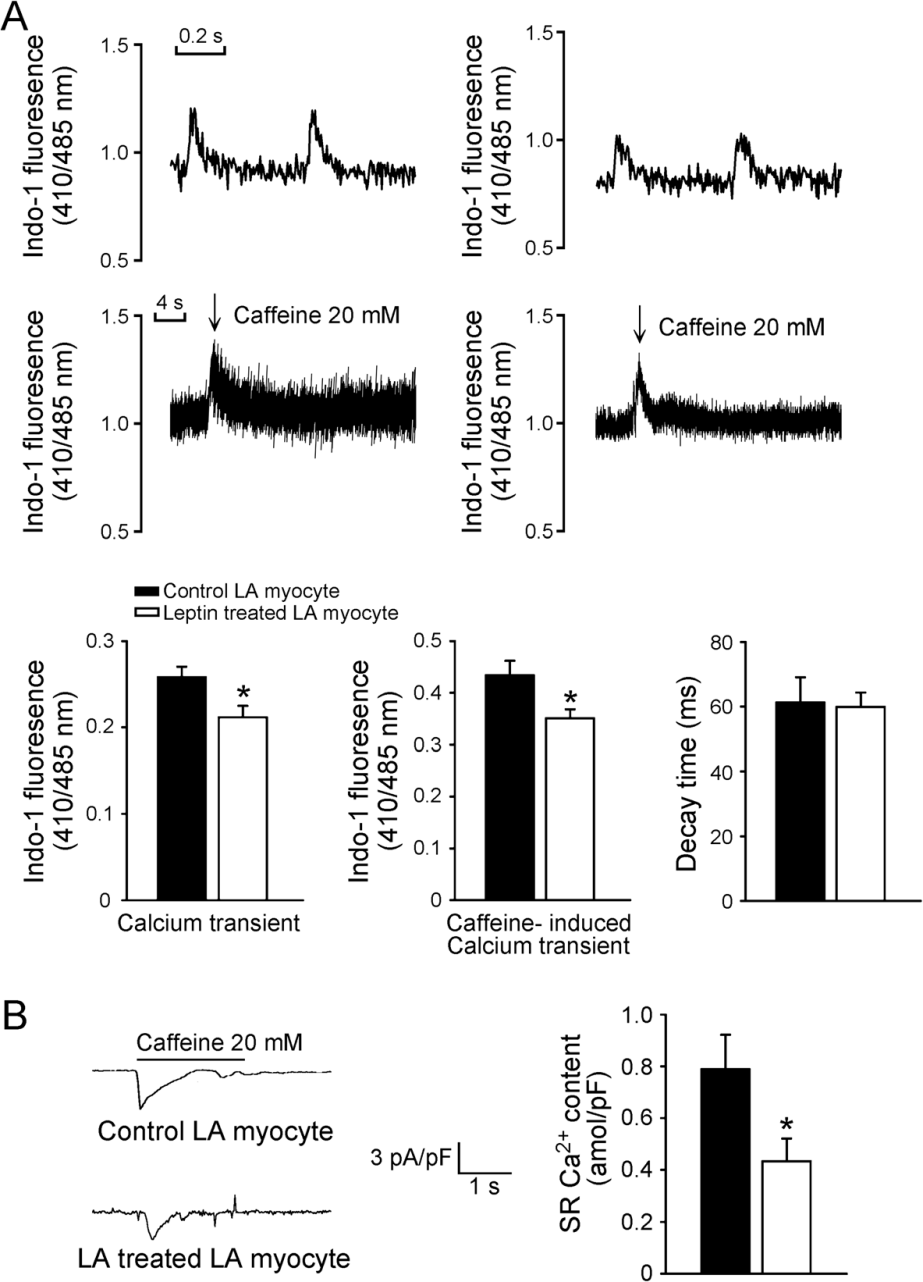
**Effects of leptin on the intracellular Ca**^**2+ **^**and sarcoplasmic reticulum Ca**^**2+ **^**content of the LA myocytes. A**. The tracings and average data from the Ca^2+^ transient and caffeine-induced Ca^2+^ transient in the control (n = 42) and leptin-treated LA myocytes (n = 38). **B**. The tracings of the caffeine-induced NCX currents and average data of SR Ca^2+^ content from integrating the NCX current in the control (n = 15) and leptin-treated LA myocytes (n = 11). *p < 0.05 versus the control.

As shown in Figure 6A, the leptin-treated LA myocytes exhibited a smaller amplitude of Ca^2+^_*i*_ transients than the control myocytes. In addition, the leptin-treated LA myocytes exhibited a smaller caffeine-induced Ca^2+^_*i*_ transients than the control myocytes, which was in consistent with a reduced sarcoplasmic reticulum (SR) Ca^2+^ content from integrating caffeine-induced NCX current in the leptin-treated LA myocytes (Figure 6B).

## Discussion

Obesity is characterized by leptin resistance, which is additionally associated with chamber dilatation, dysfunction and increased risk of heart failure [33]. Adipose tissue is a large endocrine organ that secretes numerous adipokines. The effects of leptin on the heart are more pronounced in cases of metabolic syndrome or obesity, where it primarily acts systemically in visceral fat or acts locally through either direct interactions or through paracrine mechanisms in pericardial fat [10-12]. Leptin, one of the adipokines, is secreted by adipocytes as well as by cardiomyocytes and endothelial cells [13-16]. Leptin may regulate the physiology of the heart including myocyte contractility and metabolism [30], which both influences cardiac electrophysiology.

In this study, we found that leptin-treated LA myocytes had a longer APD_20_ and a shorter ∆APD_90-20_. The inhomogeneously prolonged AP duration (triangulation) had been proposed as an arrhythmogenic factor [34]. Therefore, the increase of APD_20_ and the decrease of ∆APD_90-20_ may provide an anti-arrhythmic effect by reverse-triangulation of AP. Additionally, as compared to the control, we observed that leptin- treated LA myocytes exhibited a lower incidence of isoproterenol-induced DAD than the control did, which suggests that leptin has potential anti-arrhythmic effects. Isoproterneol has been shown to increase several potassium currents [35-37], which can result in the shortening of AP duration in control and leptin-treated myocytes. However, a previous study had revealed that epicardial fat can prolong the AP duration, and positively shift RMP, and increased isoproterenol-induced arrhythmogenesis [11]. Our findings suggest that leptin may not be responsible for arrhythmogenesis of adipose tissue. In contrast, we propose that leptin attenuates the effects of adipose arrhythmogenesis. Obesity amplifies the production of multiple adipokines, and it is possible that adipokines may offset the anti-arrhythmogeneic potentials of leptin, thereby increasing the risks of AF in obesity. In addition, leptin resistance has been suggested to impair the beneficial effects of leptin [38].

We found that the *I*_Na_ current density in the leptin-treated LA myocytes was significantly larger than that of the control LA myocytes. Increasing fast *I*_Na_ may improve conduction properties in cardiac cells, and thus reduce the risk of conduction block [39]. In contrast, the *I*_Na-Late_ was similar between the control and leptin-treated LA myocytes. *I*_Na-Late_ was demonstrated to play a vital role in the arrhythmogenic potentials of the atria [40]. The larger *I*_Na_ but similar *I*_Na-Late_ in leptin-treated LA myocytes suggests a favorable role of leptin on atrial arrhythmogenesis.

NCX plays a critical role in the atrial arrhythmogenesis [41]. In this study, we found that leptin-treated LA myocytes exhibited reduced NCX. The leptin-treated LA myocytes also exhibited smaller Ca^2+^_*i*_ transient and smaller SR content than the control myocytes, which was in consistent with the known effects of leptin on ventricular myocytes [30]. The observed Ca^2+^_*i*_ transient and SR Ca^2+^ content reduction in leptin-treated LA myocytes may be caused by the suppression of NCX with leptin. Leptin has been shown to increase the production of nitric oxide [42], which exerts cardiac-depressive actions. Therefore, the nitric oxide-mediated electrophysiological effect plays a role in leptin’s reduction of Ca^2+^_*i*_ transient and SR Ca^2+^ content. Furthermore, the effects of leptin on calcium regulations may attenuate the isoproterenol-induced arrhythmogenesis by decreasing the genesis of the DAD.

*I*_Kur_ are atrial specific ionic currents and plays an important role in atrial arrhythmogenesis [43]. We found that the leptin-treated LA exhibited smaller *I*_Kur_, which may produce a larger APD_20_ and a smaller ∆APD_90-20_. These effects may also contribute to an anti-AF potential. However, the *I*_K1_ was similar between the control and the leptin-treated LA myocytes, which may account for the similar RMP in these cells. Although adult rat ventricular myocytes incubated with leptin (100 ng/ml) for 48 hours have been shown to increase the expressions of *I*_to_ α-subunits (Kv4.2, Kv4.3) and Kv channel interacting protein (KChIP2), this study found that incubation of leptin at 100 nM for a short period did not change *I*_to_ in atrial myocytes [44].

### Potential limitations

The data in this study should be interpreted with caution because of its potential limitations. First, similar to those in the previous studies, this short-period experiment has used a supra-physiological concentration (100 nM) of leptin, which is higher than the clinically relevant concentrations [30]. The supra-physiological level of leptin may potentially reduce body weight through systemic administration of leptin in animal experiments [45,46]. Second, the incubation time was only 1 h, which represents an acute effect of leptin. A longer incubation time might yield distinct results. In addition, only healthy atria tissue was used in this study, the results may differ in diseased atrial tissues. Finally, the underling molecular mechanisms for the electrophysiological effects were not elucidated in this study. JAK/STAT signaling had been suggested to play a role in the cardiac effects of leptin [47,48]. However, it is not clear whether the prolongation of APD_20_ or the increase of *I*_Na_ can be blocked by JAK2 inhibition.

## Conclusions

Leptin regulates the LA electrophysiological characteristics and calcium homeostasis. Leptin attenuates the effect of isoproterenol-induced arrhythmogenesis, which may play a favorable role in the pathophysiology of atrial arrhythmogenesis.

## Competing interests

The authors declare that they have no competing interests.

## Authors’ contributions

YKL interpreted the data and drafted the manuscript. YCC, JHH, and YJL performed the experiments and revised it for scientific content. SSH, SAC and YJC conceived of this study, and participated in its design and coordination. All authors read and approved the final manuscript.
